# Scale-up of Digital Innovations in Health Care: Expert Commentary on Enablers and Barriers

**DOI:** 10.2196/24582

**Published:** 2022-03-11

**Authors:** Hannes Schlieter, Lisa A Marsch, Diane Whitehouse, Lena Otto, Ana Rita Londral, Gisbert Wilhelm Teepe, Martin Benedict, Joseph Ollier, Tom Ulmer, Nathalie Gasser, Sabine Ultsch, Bastian Wollschlaeger, Tobias Kowatsch

**Affiliations:** 1 Research Group Digital Health Faculty of Business and Economics Technische Universität Dresden Dresden Germany; 2 Center for Technology and Behavioral Health Geisel School of Medicine Dartmouth College Lebanon, NH United States; 3 European Health Telematics Association Brussels Belgium; 4 Value for Health CoLAB NOVA Medical School Nova University of Lisbon Lisbon Portugal; 5 Comprehensive Health Research Center NOVA Medical School Nova University of Lisbon Lisbon Portugal; 6 Centre for Digital Health Interventions Department of Management, Technology and Economics ETH Zurich Zurich Switzerland; 7 Institute of Information and Process Management Eastern Switzerland University of Applied Sciences St. Gallen Switzerland; 8 Swiss Lung Association Bern Switzerland; 9 Centre for Digital Health Interventions Institute of Technology Management University of St. Gallen St. Gallen Switzerland

**Keywords:** digital health, health care delivery, health interventions, digital health services, enablers, barriers

## Abstract

Health care delivery is undergoing a rapid change from traditional processes toward the use of digital health interventions and personalized medicine. This movement has been accelerated by the COVID-19 crisis as a response to the need to guarantee access to health care services while reducing the risk of contagion. Digital health scale-up is now also vital to achieve population-wide impact: it will only accomplish sustainable effects if and when deployed into regular health care delivery services. The question of how sustainable digital health scale-up can be successfully achieved has, however, not yet been sufficiently resolved. This paper identifies and discusses enablers and barriers for scaling up digital health innovations. The results discussed in this paper were gathered by scientists and representatives of public bodies as well as patient organizations at an international workshop on scaling up digital health innovations. Results are explored in the context of prior research and implications for future work in achieving large-scale implementations that will benefit the population as a whole.

## Introduction

Health care delivery is undergoing a rapid change from traditional processes toward the use of digital health services [1-3], that is, “tools and services that use information and communication technologies to improve prevention, diagnosis, treatment, monitoring, and management of health-related issues and to monitor and manage lifestyle habits that impact health” [4]. With this shift also comes a move to precision medicine [5] and precision health [6]. Hospitals and health care providers introduce hospital information systems [7,8], electronic health records [9-11], and telemedicine solutions for more efficient workflows within and beyond institutions [12,13]. Many people are choosing among a wide range of digital health services provided by wearables and mobile phone apps that support their self-management, health, and well-being [14]. These technologies may increasingly employ digital biomarkers to sense states of vulnerability [15,16], text- or voice-based conversational agents for intervention delivery [17-19], or a mixture of human and digital support via blended treatments [20,21]. These digital health services may be able to intervene with the right type of support, at the right time, while including contextual factors that offer a distinct contribution outside of human-delivered care [22]. Even though the number of existing services is growing, not many users are currently taking full advantage of these services. However, a better adoption by users would facilitate the diffusion of these innovative services [23]. The question of how sustainable digital health diffusion can be successfully achieved by scaling up individual services, that is, reaching more people benefitting from them [24], is not sufficiently solved yet, despite plenty of theoretical insights being available. To also consider a practical perspective, experts of digital health services gathered at a workshop. The results of the workshop combined practice- and research-based perspectives, which were matched with theoretical insights in this paper. Therefore, this paper goes one step further toward proposing a solution for the issue of scaling up digital health innovations by identifying and discussing barriers and enablers from a holistic point of view. Owing to the special circumstances of the workshop being held right at the onset of the COVID-19 pandemic in Europe, a discussion on how these barriers and enablers were affected by the COVID-19 crisis is also provided.

### Workshop Context and Contents

Barriers and enablers for scaling up digital health innovations were identified and discussed in the context of a conference workshop. The workshop participants, who are also coauthors of this paper, were both junior (n=8) and senior scientists (n=2) and representatives from nongovernmental organizations (n=2) and a home care provider (n=1). The participants came from diverse countries with backgrounds in public health, implementation science, information systems research, and computer science. Many of the participants had several years of experience with the design and implementation of digital health services. All participants came together at the “1st International Workshop on Best Practices for Scaling-Up Digital Innovations in Health care–Scale-IT-up!” The workshop was held at the BIOSTEC conference in Valletta, Malta, on February 25, 2020 and spanned 3 sessions, with 5 papers presented by some of the workshop’s participants [25-29]. Two keynote speeches on digital health innovations were given by Lisa A Marsch from the Dartmouth College in the United States and Diane Whitehouse from the European Health Telematics Association in Belgium, who both have extensive experience in scaling up digital health innovations. For example, Dr Marsch codeveloped the most empirically supported digital behavioral therapy for substance use disorders: it became the very first “prescription digital therapeutic” approved in the United States by the Food and Drug Administration [30-32]. Diane Whitehouse, as principal eHealth consultant in European Health Telematics Association, has followed a range of scaling-up projects. Examples include one related to telemedicine [33] and another related to integrated care [34]. Based on this experience, both keynotes presented insights from various international initiatives and projects. With the agreement of the speakers, the presentations and keynotes have been made available to the general public [35]. Each presentation provided a different focus on what drives the successful scale-up of digital health innovations: for example, how financial incentives need to be defined to motivate patients to adopt digital health innovations successfully. Based on the input of all presenters, the last workshop session featured a discussion of all 13 speakers and workshop participants on best practices and challenges for scaling up digital health innovations. This last workshop session forms the basis for the method used to identify the relevant set of enablers and barriers.

## Methods

The discussion during the last session of the workshop followed a structured approach. To this end, 2 topic leaders—one each for the topics of barriers and enablers—were determined. The remaining participants were then split into 2 groups. Both groups undertook a 2-round group process, with discussions on barriers and enablers.

In the first round, the groups identified either enablers or barriers for scaling up digital health innovations depending on their initial topic assignment. This identification was carried out according to the brainwriting technique [36], with each participant writing down items individually. This process enabled the participants to take their time and to be equally involved in the process. Afterwards, each participant presented and explained their list of enablers or barriers to the group so that the ideas could be consolidated and clustered. This process took 20 minutes before group members switched to the other topic (ie, from enablers to barriers and vice versa). The 2 topic leaders remained to inform the other group members about the intermediate results.

In the second round, the other group was informed about the results of the first round and could extend and revise these findings. Finally, all participants were given the final results, which were discussed and consolidated until a group consensus between all 13 participants was reached. Owing to our clustering, some items could have been mentioned multiple times and therefore carried more weight than others. However, no weights were added after identifying and discussing the findings. Afterwards, the raw results of each topic were digitized and categorized, and duplicates were removed. Finally, the results were aggregated and aligned with the existing work of DeLone and McLean [37] regarding enablers and Kowatsch et al [3] regarding both enablers and barriers. This allowed us to validate the results gained and to assess whether new aspects have been identified.

## Results

In total, 36 enablers and 33 barriers were identified in the workshop session. To align these enablers and barriers with prior research, they were grouped in categories classified by DeLone and McLean [37] as enablers and Kowatsch et al [3] as both enablers and barriers. To further understand the context of digital innovations in health care, the aspects were also grouped according to 5 various levels of influence [38], namely, the micro, meso, macro, and the technology/innovation level, or in an overarching category. Some barriers and enablers cannot be influenced by only one of the 4 levels and were therefore grouped into an overarching category.

Overarching aspects that are influenced by all stakeholders involved on all levels are leadership, culture, interdisciplinary cocreation, innovation characteristics, and methodology. Although missing leadership in projects is an exemplary barrier hindering the scale-up of digital health innovations, dialogue between all stakeholders involved can be supportive. Further, a culture in favor of digital health innovations needs to exist, as it otherwise hinders the scale-up process when not existent. If interdisciplinary cocreation is missing, this can be a barrier, while collaboration between stakeholders or stakeholder engagement are exemplary enablers: indeed, the importance of the “quadruple helix” [39] and engagement cannot be understated. Characteristics of the innovation itself and the methodology mainly act as barriers for digital health innovations. Participants referred to a lack of trust when transferring existing solutions to new contexts (the “not invented here” dilemma). Named as a further potential barrier, a pace of multistakeholder innovation that is too high may lead to piecemeal approaches or to not paying heed to past successes/failures in other settings when designing new approaches. All overarching barriers and enablers are presented in Table 1.

**Table 1:** Overarching enablers and barriers for scaling up digital health innovations identified in the workshop and aligned with prior work.

Category	Enabler	Barrier
Leadership^a^	Continuous dialogue between academia, industry, government, and other stakeholders to facilitate policy-relevant research and increase scale-up of science-based best practices Visionary leadership: clear idea of leadership what the digital health innovation should look like in the future Care management: managing medical conditions more effectively by patient-centered approach that is designed to assist patients and their support system	Missing leadership in projects
User culture^b^	—^c^	Inherent characteristics and preferences of specific user groups
Interdisciplinary cocreation^a^	Collaboration between medical experts, computer scientists, business experts, etc Continuous dialogue between academia, industry, government, and other stakeholders to facilitate policy-relevant research and increase scale-up of scientifically validated best practices Employee involvement (direct participation of staff, eg, applying own ideas, expertise, efforts for developing digital health interventions) Engagement of diverse stakeholders/stakeholder engagement (involving stakeholders in the systematic identification, analysis, planning, and implementation of actions for the digital innovation)	Missing cocreation (designers of digital innovation do not include medical, information technology, and business staff) Gap between technology developers/researchers and health care practice (involved parties have an opposing understanding or underestimate the time needed to complete relevant steps) Knowledge in silos (data and document systems of the digital innovation are not shared between all people involved) Missing common goal (goals of different stakeholders do not align)
Innovation characteristics^a^	—	Who pays the risk of innovation? (liability issues and uncertainties when digital health innovations are integrated into the treatment process) Too high pace of technology inventions (many technical improvements in a short time making it difficult to implement them owing to a steady flow of new technologies) Need for speed (rapidity/pressure of change) The “not invented here” dilemma (successful and effective digital innovations are not implemented when users or clinicians were not involved)
Methodology^b^	—	Selection bias (certain types of professionals or clinicians are more interested in developing digital innovations; furthermore, certain users may be more eager to participate and use digital innovations)

^a^Additional category based on conclusions from the workshop.

^b^Category adopted from Kowatsch et al [3].

^c^Not available.

Barriers rather than enablers were identified on the macro level, referring to legislation, regulation or finance guidelines, and the respective stakeholders. The characteristics of regulation, funding, reimbursement, and planning are currently mainly hindering a successful scale-up of digital health innovations. Liability issues or the generally missing innovation-friendliness in the health care system together with missing funding or reimbursement can be named as examples. Further, the experts shared their experience that the aim of most research is not a successful implementation. All barriers and enablers on the macro level are presented in Table 2.

The meso level, for example, the community around individual end users, on the contrary, seems to enable the scale-up of digital health innovations rather than hinder it. Even though using different infrastructure systems as regional infrastructure can be a barrier, culture and social support can help in raising awareness and building capacity and trust. All barriers and enablers on the meso level are presented in Table 3.

**Table 2:** Enablers and barriers for scaling up digital health innovations on the macro level identified in the workshop and aligned with prior work.

Category^a^	Enabler	Barrier
Regulatory issues	A method for approval of market entry (guidelines and rules along which new digital innovations can be developed and introduced, eg, Digital Health Applications process from the German Federal Institute for Drugs and Medical Devices) [40] Legislative change (recent changes improving the development, implementation, and reimbursement of digital health innovations, eg, Digital Healthcare Act [Digitale-Versorgung-Gesetzes] passed on December 19, 2019, the “app on prescription” for patients was introduced into health care [sections §§ 33a and 139e of the Fifth Book of the German Social Code Book V])	Legal regulations (legislation that regulates development and market entry for digital health innovations) Liability issues (unclear who is responsible for the safety and possible claims that could arise in the future) High regulatory barriers (overhead to develop and distribute digital innovations is high) Health system is not innovation-friendly
Funding	—^b^	Missing funding (funding necessary for the development and accreditation of digital health innovations is often missing)
Reimbursement	—	Reimbursement is not guaranteed (unclear whether health care providers will get paid for using/prescribing the digital health innovation)
Planning	—	The aim of the research is not a successful implementation

^a^All categories were adopted from Kowatsch et al [3].

^b^Not available.

**Table 3:** Enablers and barriers for scaling up digital health innovations on the meso level identified in the workshop and aligned with prior work.

Category^a^	Enabler	Barrier
Regional infrastructure	—^b^	Different infrastructure systems are used (eg, different database, digital health records, diagnosis, and treatment codes)
Culture	Organizational change (change from one state of affairs to another in the form of, eg, company’s structure, strategy, policies, procedures, technology, or culture) Capacity building (obtaining, improving, and retaining the skills knowledge, tools, equipment, and other resources needed to achieve organizations’ goals) Awareness raising (inform and educate individuals about a topic or issue with the intent to change their attitudes, behaviors and beliefs, eg, the potential of digital innovations for health) Prioritization of trustworthy digital health (professionals and users need to trust in the digital health innovations’ effectiveness and safety)	—
Social support	Trust building (increasing the users’ trust in the safety and effectiveness as well as the vision and mission of the digital innovation and the individuals or organizations involved)	—

^a^All categories were adopted from Kowatsch et al [3].

^b^Not available.

On the micro level, the level of individual end users, the recommendation of digital health innovations by physicians (social support) can enable their scale-up. Other individual characteristics of the end users, for example, lacking motivation or trust, their individual resources, or negative associations are rather hindering. The latter two mainly refer to the physicians/professionals as end users as they could be affected by additional work or see digital health innovations as a threat, negatively influencing their scale-up. All barriers and enablers on the micro level are presented in Table 4.

Technical aspects, particularly aspects regarding the innovation itself, form the largest group of enablers and barriers named by the experts. The categories related are information quality, usability, integration, interoperability, the business model, standards, and the innovation process itself. Although information quality (eg, open source) is seen as an enabler, usability is only seen as a barrier if not well-thought-out (eg, lack of ease of use, too high complexity). Integration and interoperability aspects can hinder the innovation if not sufficiently considered but can also support actively the innovation’s success. Providing incentives or added value and having the business model in mind already at an early stage are important aspects for successfully scaling up digital health interventions. If, on the contrary, no suitable business model or no sufficient value propositions exist, it can easily turn into a hindering factor. Standards are a category, which only supports the scale-up of digital health innovations if appropriately followed. Using existing infrastructure and aligning to existing standards can be an advantage of each digital health innovation. Flexibility and modularization in the innovation process can support the innovation’s success too, while the pressure of change and the risk of innovations are hindering in some cases. All barriers and enablers related to technical aspects or regarding innovation itself are presented in Table 5.

**Table 4:** Enablers and barriers for scaling up digital health innovations on the micro level identified in the workshop and aligned with prior work.

Category^a^	Enabler	Barrier
Social support	Recommendation of the digital health innovation by physicians	—^b^
Individual characteristics of end user	—	Lack of motivation to change/adapt (digital innovation fails to elicit behavior) Trust issues (users do not trust the digital innovation to be effective, safe, or useful) Additional work for medical staff (digital innovation does not facilitate but increase the workload of already busy health care professionals)
Negative associations	—	Physicians perceive digital health innovations as a threat/substitution (digital health innovations as a potential replacement or restriction of professional latitude)

^a^All categories were adopted from Kowatsch et al [3].

^b^Not available.

**Table 5:** Enablers and barriers related to technical aspects or regarding innovation itself for scaling up digital health innovations identified in the workshop and aligned with prior work.

Category	Enabler	Barrier
Information quality^a^	Open source (source code of the digital innovations is made available for possible use, modification, and redistribution) Continuous clinical validation of digital innovations Information disclosure (the degree to what sensitive information is properly protected by the digital innovation) Evidence-based intervention components (components such as techniques, methods, and means to change behaviors or outcomes are based on empirical evidence) Access to patient data, software, etc (digital innovations need data from patients, access to software to be developed and validated on a larger scale)	—^b^
Usability of technology^c^	—	Lack of ease of use (digital innovation is burdensome) Complexity is too high (digital innovation targets too many outcomes or behaviors or is poorly designed) No user-centered design
Integration^c^	Integration in existing workflows (digital health innovations can be integrated into existing systems for prevention or treatment)	Integration issues (digital innovations cannot or are difficult to integrate into existing systems or workflows)
Interoperability^c^	Complemented and extended health care service delivery and research (does not compete with or disrupt workflow) Early steps on interoperability (digital innovations can exchange and use information and data from other sources)	Incompatibility of existing processes and innovation (digital innovations do not solve a problem in the current health care setting and cannot be used in other settings) Closed systems/missing interoperability (digital innovations are limited or cannot exchange and use information and data from other sources)
Business model^d^	Appropriate incentives (momentary or other compensations for participation in programs or using digital innovations) Financially viable business model (the degree to which digital innovations can cover costs and potentially generate revenue) Business model in mind at an early stage (of developing the digital health innovation) Providing added value (the degree to which the digital innovation can address the needs or ease the pains of users)	No suitable business model for preventive interventions Missing value proposition for patients (digital innovation does not solve or facilitate a problem or need of the patients)
Standards^c^	Alignment to existing standards (digital health innovation was developed by taking existing standards or guidelines into consideration) Usage of existing infrastructure (digital innovations are designed to use existing resources or incorporate relevant health care professionals) Utilization of existing organizations (patient organizations or research institutions are consulted when the digital innovation is designed)	—
Innovation process^d^	Minimum viable product and small iterations (small but working prototypes to be evaluated in continuous evaluation by users, health care providers, and health care professionals alike) Adoption, iteration, refinement, and removal of elements that do not add value Modularization regarding upscaling (further modules that extend the digital health innovation are developed and released) Flexibility in the innovation process (adjustment to findings from research and the design process are integrated) User-centered design and evaluation at every stage	Unclear/not defined process to innovate (iteration of different stages of the digital health innovation or digital health innovations, in general, is not planned)
Interdisciplinary cocreation^d^	Patient inclusion (patients are integrated into each step when designing and evaluating the digital health innovation)	Missing broad stakeholder engagement (focusing on only one group)

^a^Category adopted from DeLone and McLean [37].

^b^Not available.

^c^Category adopted from Kowatsch et al [3].

^d^Additional category based on conclusions from the workshop.

## Discussion

Workshop outcomes were used to compile a classified list of enablers and barriers. The workshop’s aim was to match existing theoretical insights on enablers and barriers of digital health innovations with the practical experiences that the workshop participants brought to the activity. Participants offered insights from both research-based (empirical and applied) and real-life perspectives. After the workshop, the resulting brainstorming (brainwriting) results were classified into categories to reach a single consolidated list of enablers and barriers. The results represent the collective perspective of the group members who participated in the working session. The viewpoint represented is that of a set of people who have actively participated in different forms of research on the development and implementation of digital health interventions.

The categories of enablers and barriers have shown that the successful scale-up of digital health innovations is influenced by actors and aspects on different levels (micro, meso, macro, and technology/innovation level). Actors in each of these levels are perceived to influence the success of digital health innovations. These different levels are in line with the focus areas that were identified by Labrique et al [41] critical for scaling digital health initiatives: health care ecosystem (macro level), extrinsic ecosystem (meso level), intrinsic characteristics, human factors (micro level), and technical factors (technology/innovation). Our viewpoint contributed to further specify, in each level, enablers and barriers experienced by different stakeholders.

Prior work of some of the participants in the workshop [3] covers diverse categories of influencing factors on digital health innovations. Although most of these categories were mirrored by the results of the workshop, some were not named at all by the workshop participants, as is the example of the disease, social interaction, or expectations. Other categories such as standards or social support were only named as enablers even though they also represent barriers according to Kowatsch et al [3]. However, it is a noteworthy fact that some of the aspects pointed out by the workshop participants add a new contribution to our prior work, namely, the characteristics and the process of innovations, leadership, interdisciplinary cocreation, and the business model.

Leadership was referred to as both enabler and barrier (when considering the lack of it). It is an essential trigger for digital innovation and adoption [42]. In line with leadership, the culture for change, the need for common goals, prioritization, and planning were mentioned in the workshop as relevant for scale-up. These relate to the intimate connection between digital health innovation and the changes that it drives in delivering health care services [43]. Scaling up digital health must be driven by synergic interventions in health care services and workflows. This justifies the cocreation interdisciplinary category mentioned in the workshop, reinforcing the need of the different stakeholders to be engaged and to be considered. Cresswell et al [44] suggest 10 key considerations for the case of health information technology, where including professional, administrative, and managerial teams to define the needs and build consensus around a strategic vision is key to successfully implement technology. A less siloed approach that motivates interdisciplinary cocreation is referred to in both our workshop findings and the literature [41].

Further, commonly mentioned in the workshop, at different levels, are the regulatory and trust issues that hamper adoption at a large scale. Indeed, policymakers and health care management need to be part of innovation processes, understand the needs for change, and provide guidelines that increase trust to take decisions on scaling up [42]. Business models were also mentioned in our workshop as a category that is relevant for scaling up. This relates to the need to develop a multistakeholder perspective on value delivery in the health care ecosystem [45]. Further research should develop guidelines that consider the different levels and categories that were discussed in this paper to support technology innovators and providers in planning the scale-up of digital health innovations.

It needs to be elaborated further if the discrepancy between the findings of our workshop and prior literature shows different perspectives in the perception of enablers and barriers for digital health innovations between research and practice. Moreover, future research should corroborate our findings with larger groups of experts to see how they hold or vary per medical subindustry. We suggest that the development of digital health apps for smartphones may be different from the development of hospital information systems. The findings reported are mainly limited by the size of the expert group involved in the workshop. It could have been too small or not representative enough since the group was composed mainly of researchers. Nevertheless, the experts involved combined extensive experience in (digital) health care.

### Importance of COVID-19 to Scale-up

The workshop took place at the very onset of the pandemic breakout; days after it occurred, Malta restricted travel in and out of the island. As so, the reported viewpoint did not consider all the digital transformation that happened in the last year owing to the need for fast response to health care needs. Many identified barriers for adoption and scale-up were suddenly put aside owing to the urgent need to provide remote and safe care and monitoring. Leadership, regulatory issues, and reimbursement are categories of those barriers, which were also identified in Table 1. Governments incentivized telecare as a digital-first pathway, followed by a rapid change in the insurance companies that reimbursed teleconsultations. Many countries relaxed privacy and data protection regulations during the crisis under General Data Protection Regulation exceptions for public interest [46]. Cooperation and evidence of the benefits for the patients were enablers, identified in this viewpoint, for the fast scale-up and adoption of digital health. However, digital health interventions played a marginal role owing to the inadequacy of protocols and lack of readiness for implementation [47], which may be mainly related to the categories of barriers identified in Table 1: innovation characteristics, planning, integration, and culture. Digital adoption by health care was held by those technologies that were already mature, commonly used and that could be integrated into existing workflows. One example was the massive adoption of videocall platforms and instant messaging apps that were provided for teleconsultations as urgent replacements for usual clinical consultations to respond to the population questions and concerns of the general public [48]. In addition to changes taking place in health care organizations, citizens themselves scaled up the adoption of smart devices that were already on the market [49,50]. Symptom-checking and contact-tracing apps were downloaded to millions of smartphones under the polemic of data privacy versus population safety [51]. This may be related to increased motivation and trust by the population, listed as enablers at a micro level in Table 1. The COVID-19 pandemic has led to a rapid scale-up of telehealth services. Two strong enablers included the urgent and immediate demand from health and care systems as well as the population at large and the readiness of global technology companies that were ready to adapt their technologies to respond to the needs of the context. In the crisis, these enablers demonstrated how one can, in reality, suspend barriers that have previously been identified as causes of delay in digital adoption [52]. Although some of the barriers identified in this viewpoint may have been reduced (eg, lack of motivation to change), others were simply ignored temporarily and remain to be tackled in the future (eg, legal regulations).

### Conclusions

To summarize the findings and discussions from the workshop, 5 conclusions for the scale-up of digital health innovations can be highlighted. First, digital health services can help to drive data quality, outreach to communities, and manage disease transmission/progression. Second, to reach these aims, a general cultural shift is needed when aiming to have digital services as viable instruments in health care along with classic pharmaceutical, surgical, or other therapeutic measures. Third, technological developments and interoperability appear to be enablers supporting digital health services rather than acting as hindrances. This latter finding is rather surprising since lack of interoperability has often been named as a barrier in prior work [53,54]. Indeed, the European Commission has long called for a more extensive focus on interoperability to facilitate the increased use of digital health technologies. Fourth, when scaling up digital health innovations, it is important to ensure the involvement of all stakeholders, including people from different professions and occupations, and especially patients and citizens. Only through a joint effort on the part of all stakeholders can digital health services succeed. Fifth, the innovation process itself also plays a crucial role, especially in relation to culture and leadership. The innovation process should be partitioned into different stages. Within each stage, further research should examine how to best fulfill the respective stage. Innovation processes should also be considered in reimbursement models for digital health innovations to ensure that new technologies such as digital pills have a chance to be tested in real-world settings. When working on all the 5 aspects of enablers and barriers to digital innovation in health and care, we believe that the scale-up of digital health services can be strongly supported. A clear view of these aspects would guide the application of the growing funding for health care information technology toward accelerating its impact in health care services.
